# The Surface Amine Group of Ultrasmall Magnetic Iron Oxide Nanoparticles Produce Analgesia in the Spinal Cord and Decrease Long-Term Potentiation

**DOI:** 10.3390/pharmaceutics14020366

**Published:** 2022-02-06

**Authors:** Guan-Ling Lu, Ya-Chi Lin, Ping-Ching Wu, Yen-Chin Liu

**Affiliations:** 1 Department of Anesthesiology, School of Post-Baccalaureate, College of Medicine, Kaohsiung Medical University, Kaohsiung 807, Taiwan; r93443013@ntu.edu.tw; 2 Department of Anesthesiology, National Cheng Kung University Hospital, College of Medicine, National Cheng Kung University, Tainan 701, Taiwan; tonyangiekiki@hotmail.com; 3 Department of Biomedical Engineering, National Cheng Kung University, Tainan 701, Taiwan; 4 Institute of Oral Medicine and Department of Stomatology, National Cheng Kung University Hospital, College of Medicine, National Cheng Kung University, Tainan 701, Taiwan; 5 Center of Applied Nanomedicine, National Cheng Kung University, Tainan 701, Taiwan; 6 Medical Device Innovation Center, Taiwan Innovation Center of Medical Devices and Technology, National Cheng Kung University Hospital, National Cheng Kung University, Tainan 701, Taiwan; 7 Department of Anesthesiology, Kaohsiung Medical University Hospital, Kaohsiung 807, Taiwan

**Keywords:** ultrasmall magnetic iron oxide nanoparticles, inflammatory pain, analgesia, pro-inflammatory cytokines, neurotoxicity, long-term potentiation

## Abstract

Our previous studies have revealed the ultrasmall superparamagnetic iron oxide in the amine group USPIO-101 has an analgesic effect on inflammatory pain. Here, we further investigated its effect on the spinal cord and brain via electrophysiological and molecular methods. We used a mouse inflammatory pain model, induced by complete Freund’s adjuvant (CFA), and measured pain thresholds via von Frey methods. We also investigated the effects of USPIO-101 via an extracellular electrophysiological recording at the spinal dorsal horn synapses and hippocampal Schaffer collateral-CA1 synapses, respectively. The mRNA expression of pro-inflammatory cytokines was detected by quantitative real-time polymerase chain reaction (RT-qPCR). Our results showed intrathecal USPIO-101 produces similar analgesic behavior in mice with chronic inflammatory pain via intrathecal or intraplantar administration. The potentiated low-frequency stimulation-induced spinal cord long-term potentiation (LTP) at the spinal cord superficial dorsal horn synapses could decrease via USPIO-101 in mice with chronic inflammatory pain. However, the mRNA expression of cyclooxygenase-2 was enhanced with lipopolysaccharide (LPS) stimulation in microglial cells, and we also found USPIO-101 at 30 µg/mL could decrease the magnitude of hippocampal LTP. These findings revealed that intrathecal USPIO-101 presented an analgesia effect at the spinal cord level, but had neurotoxicity risk at higher doses.

## 1. Introduction

The therapeutic application of iron oxide nanoparticles has been developing over the years. Currently, the commercialized product of iron oxide nanoparticles are used for the treatment of cancer and iron-deficiency anemia [1,2]; however, a lesser known use for iron oxide nanoparticles is pain management. The ongoing nanoparticle-based therapeutics in pain management [3] have several advantages for chronic pain relief, for example, controlled release, prolonged circulation time, and limited side effects [4].

Our previous study revealed a form of amine-terminated (-NH_2_) ultrasmall superparamagnetic iron oxide (USPIO) called USPIO-101, which has an analgesic effect on inflammatory pain [5]. This analgesic response probably happens by attenuating inflammatory cell infiltration and reducing reactive oxygen species (ROS) production in the paw [5]. However, this was the first report to demonstrate that USPIO itself could have analgesic ability not in conjugation with other pain-relieving drugs.

Spinal cord synaptic plasticity is an *in vitro*, cellular, molecular model for pain [6]. Long-term potentiation (LTP) at the superficial dorsal horn synapses could also represent the nociceptive nerve signal. On the contrary, the decreased LTP level could stand for the analgesic effect of the treated compound or anti-allodynia signal [7]. However, less is known about the analgesic effect of USPIO-101 in the spinal cord via electrophysiological evidence. Here, we use *in vitro* extracellular recording to measure the effect of USPIO-101 on the spinal LTP to demonstrate the analgesic effect of USPIO-101 on the spinal cord.

Before developing the application of USPIO-101, one crucial indication was to lower cell cytotoxicity. In addition, USPIO-101 in a small size could probably interfere with normal body function via crossing the blood–brain barrier [8]. Therefore, the cytotoxicity of USPIO-101 is unclear. However, some reports have revealed the cytotoxicity of other types of USPIO. For example, the acute intravenous (iv.) injection of USPIO caused thrombosis, cardiac oxidative stress, and DNA damage in mice [9]. Furthermore, USPIO also triggered interleukin (IL)-6-related acute-phase inflammation [10] with a mechanism of endoplasmic reticulum (ER)-mitochondria Ca^2+^ crosstalk, which was mediated by cyclooxygenase-2 (COX-2) [11] in hepatocytes. Finally, superparamagnetic iron oxide (SPIO) administration, either locally or systemically, gave an acute inflammatory response [12]. These reports inspired us to consider that USPIO-101 could probably have neurotoxicity in neuronal cells. Thus, we investigated the toxicity of USPIO-101 in neuron-like or microglial cells via measuring the ROS production or mRNA expression of pro-inflammatory cytokines in the present study.

Synaptic plasticity is fundamental to many neurobiological functions, including memory and pain [13]. Moreover, the hippocampus’s long-lasting potentiated synaptic field potentials are a proposed cellular mechanism for memory [14]. Here, we examined the toxicity effect of USPIO-101 on hippocampal LTP, which represented a higher level of neurobiological functions via *in vitro* extracellular recording at Schaffer collateral/CA1 synapses.

In this study, we revealed further analgesic evidence for using USPIO-101 at the spinal cord and the possible neurotoxicity that should be concerned for future application.

## 2. Materials and Methods

### 2.1. Drugs and Administration

The amine-terminated (-NH_2_) iron oxide nanoparticles (Fe_3_O_4_ NPs) were commercially purchased from TANBead (USPIO-101, Taiwan Advanced Nanotech Inc., Taoyuan, Taiwan), and the stock concentration was 10 mg/mL. For behavioral tests, the intrathecal or intraplantar injection, the stock solution was used in a volume of 10 μL. For *in vitro* electrophysiological study, 1000X dilution was used for perfusion.

### 2.2. The Particle Size, Zeta Potential, and Surface Group Measurement of Iron Oxide Nanoparticles

The particle size distribution and zeta potential were measured by dynamic light scattering (DLS) (Beckman Coulter DelsaTM Nano instrument, CA, USA) with deionized water (ddH_2_O) as the solvent. Fourier transform infrared (FTIR) spectra analyzed the surface group of the iron oxide nanoparticles via Nicolet FTIR spectrometers (Thermo Scientific, MA, USA) in the range 500–4000 cm^−1^ using a resolution of 1 cm^−1^ and 10 scans. In advance of testing, the particles were placed in an oven (60 °C) overnight to remove water and then ground with potassium bromide (KBr) powder to increase the absorption of infrared light and eventually pressed to obtain self-supporting discs.

### 2.3. Animal

Male ICR mice were used in all the experiments: for spinal cord slices electrophysiological recording, we used 4~6-week-old mice; others were 6~8-week-old mice. All animals were purchased from Bio-LASCO Inc. (Taipei, Taiwan). Mice were housed 4~5 per cage under 12 h light/dark controlled (AM/PM 7:00) with free access to food and water. The animals used for electrophysiological recording were housed in the NHRI laboratory animal center and approved by the NHRI laboratory animal center. The animals used for the behavioral test were housed and performed in National Cheng Kung University (NCKU) Laboratory Animal Center and approved by NCKU Medical College Animal Care Guidelines.

### 2.4. CFA Inflammatory Pain Model and Behavior Tests

CFA (complete Freund’s adjuvant; Sigma-Aldrich, Saint Louis, MO, USA) or saline 10 µL were injected into the plantar surface of the left hind paw to induce an inflammatory pain model [5]. Mice were placed in individual test boxes for the mechanical pain sensitivity test. Mice were habituated for at least two days in the testing environment daily for one hour. Before the examination, at least one hour of habituation was necessary. A series of von Frey hairs with logarithmically increasing stiffness (0.02–2.56 g, Stoelting, Wood Dale, IL, USA), perpendicular to the plantar surface of the left hind paw was applied for 1 s, until it buckled. We marked a positive response if the animal exhibited any nocifensive behaviors after removing the filament, including quick paw withdrawal, licking, or shaking the paw. The first filament was chosen to be close to the 50% withdrawal threshold. If there was no response, the next filament was a higher force; if there was a response, the next filament was a lower force. This continued until at least four readings were obtained after the first change of direction [15]. The analysis of the 50% paw withdrawal threshold was determined using the Dixon up–down method and calculated using the formula: 50% threshold (g) = 10^(X+kd)^/10^4^, where X = the value (in log units) of the final von Frey filament, k = tabular value for the response pattern (see Appendix 1 in [16]) and d = the average increment (in log units) between von Frey filaments [16].

### 2.5. Electrophysiological Recordings

#### 2.5.1. Spinal Cord Slice

Transverse spinal cord slices (350 µm) were dissected as described previously with modifications [17,18]. Mice were sacrificed with overdose isoflurane and transcardial perfusion with cold artificial cerebral spinal fluid (aCSF) immediately; then, the spinal cord was removed from the spinal column. After dissection of the spinal cord pia-arachnoid membrane in cold aCSF, the spinal cord’s lumbosacral enlargement (L1–S3) was maintained. Then, we collected spinal cord slices from the L4~L6 region with a vibratome (DTK1000, Dosaka) and equilibrated slices at room temperature for at least one hour before recording. The aCSF consisted of (mM): NaCl 117, KCl 4.5, CaCl_2_ 2.5, MgCl_2_ 1.2, NaH_2_PO_4_ 1.2, NaHCO_3_ 25 and glucose 11, and was oxygenated with 95% O_2_/5% CO_2_ (pH 7.4).

We recorded the field excitatory postsynaptic potentials (fEPSPs) at the spinal cord superficial dorsal horn synapses of the mouse spinal cord slices with a continuously perfused oxygenated aCSF at 1~2 mL/min. Glass pipettes (resistance, 5~8 MΩ) were filled with aCSF. Then, according to Terman’s report [19], we determined the position of the stimulating electrode and the recording glass pipette. First, the stimulating electrode was attached to the spinal cord slice’s dorsal root remnant; second, the recording glass pipette was placed on the superficial dorsal horn area of the spinal cord slice. Signal acquisition was measured by Multiclamp 700B amplifier (Molecular Devices) and sampled by pCLAMP 10.2 and an analog-to-digital converter (Digidata 1322A), filtered at 2~5 kHz, digitized at 10 kHz, and stored for off-line analysis.

The stimulation signals were sequentially evoked (Grass S88) for thirty seconds, once, with a 0.5-ms pulse. Low-frequency stimulation (2 Hz, 120 s) was applied to induce long-term potentiation (LTP). The baseline of fEPSP was obtained 10 min from the beginning, and the slope of fEPSPs was normalized by the calculated average slope of 20 fEPSPs. The magnitude of LTP was the average slope of the last 20 fEPSPs recorded after high-frequency stimulation for 30 to 40 min. Each fEPSP was collected and analyzed by Clampfit software. Every mouse used 1~3 spinal cord slices for recording.

#### 2.5.2. Hippocampal Slice

Coronal hippocampal slices (400 µm) were dissected as described previously with modifications [20]. Mice were sacrificed with overdose isoflurane and decapitated immediately, then the brain was transferred to cold aCSF. After dissection, slices were equilibrated at room temperature for two hours before recording.

The recorded fEPSPs were evoked on the Schaffer collateral fiber path and detected in the apical dendritic field (the stratum radiatum) in the CA1 region in each hippocampal slice. Basal stimulation was given at 0.03 Hz by constant current pulses (0.2 ms). LTP was induced by theta-burst stimulation (TBS), which contained three trains of five bursts separated by 300 ms, with each burst consisting of ten pulses at 100 Hz. The baseline of fEPSP was obtained before TBS and maintained for at least 10 min. The magnitude of LTP was calculated by the average slope of 20 fEPSPs recorded after TBS 30 to 40 min. Every mouse used 1~3 brain slices for recording. Signal acquisition and analysis were similar to spinal cord slices.

### 2.6. ROS Levels

The human neuroblastoma SH-SY5Y cells, and mouse SM826 microglia cell line, were cultured in DMEM growth medium (Gibco) supplemented with 10% fetal bovine serum (FBS, BI) and 0.1% penicillin/streptomycin (Gibco). Cells were incubated at 37℃ in an atmosphere containing 5% CO_2_.

An OxiSelect intracellular ROS assay kit (Cell Biolabs, San Diego, CA, USA) was used to measure the ROS levels in the SH-SY5Y and SM826 cells, respectively. Cells were seeded into 96-well plates (4 × 10^4^ cells/well) and incubated for 16 h at 37℃. The SH-SY5Y cells were incubated with 2,7-Dichlorodihydrofluorescein diacetate (DCFH-DA) 0.05 mM/serum-free medium for 60 min at 37℃. DCFH-DA medium was removed and treated with USPIO-101 (10 or 30 μg/mL), H_2_O_2_ 1 mM for 30 min, or H_2_O_2_ 0.2 mM for 24 h stimulation. The SM826 cells were treated with USPIO-101 (10 or 30 μg/mL), or LPS (1 μg/mL) for 24 h, and then incubated with DCFH-DA (0.05 mM, 60 min)/serum-free medium. All cells had lysis buffer added 5 min before reading the fluorescence and were analyzed by a fluorometric microplate reader (SpectraMax M2) at 480 nm/530 nm.

### 2.7. Assay of mRNA Expression

Total RNA was extracted via GENEzol^TM^ TriRNA Pure Kit (Geneaid, New Taipei City, Taiwan) following the manufacturer’s instructions. Total RNA (500 ng) was utilized for the reverse-transcription polymerase chain reaction (RT-PCR) by Thermo Scientific^TM^ RevertAid RT Reverse Transcription Kit (Thermo Fisher Scientific, Waltham, MA, USA). The RNA and cDNA products were stored at -80℃ before the following experimental procedure.

The mRNA expression levels were determined by real-time quantitative polymerase chain reaction (RT-qPCR). The Applied Biosystems^TM^ StepOnePlus™ Real-Time PCR System and StepOne^TM^ Software v2.3 (Thermo Fisher Scientific, Taiwan) were used. The reagent was Thermo Scientific^TM^ Luminaris Color HiGreen qPCR Master Mix (2X) high ROX and Yellow Sample Buffer (40X) (Thermo Fisher Scientific, Waltham, MA, US). The RT-qPCR conditions were initial denaturation, 95 °C for 15 s; annealing, 60 °C for 30 s; extension, 72 °C for 30 s; 40 cycles.

The primers were synthesis from MISSION BIOTECH CO., LTD., Taiwan. The relative mRNA expression was determined by the 2^−∆∆Ct^ method using GAPDH (glyceraldehyde-3-phosphate dehydrogenase) as a normalization control.

Forward and reverse primer sets for each cDNA were used as follows: 5′-ATCTCATACCAGGAGAAAGTCAACCT-3′ and 5′-TGGGCTCATACCAGGGTTTG-3′ (for TNF-α); 5′-GCTGCCAAAGAAGGACACGACA-3′ and 5′-GGCAGGCTATTGCTCATCACAG -3′ (for NF-kB); 5′-GGCCATGGAGTGGACTTAAA-3′ and 5′-CACCTCTCCACCAATGACCT-3′ (for COX-2); 5′-TGTGTCCGTCGTGGATCTGA-3′ and 5′-GATGCCTGCTTCACCACCTT-3′ (for GAPDH).

### 2.8. Statistical Analysis

All results are expressed as the mean ± SEM (standard error of mean). Electrophysiological results, ROS level, or mRNA expression were analyzed by one-way analysis of variance (ANOVA) followed by Newman–Keuls multiple comparisons test for post-hoc analyses. Behavioral results were analyzed with repeated-measure two-way ANOVA followed by Tukey tests for post-hoc analyses. The criterion for statistical significance was *p* < 0.05 when compared with each group.

## 3. Results

### 3.1. The Particle Size, Zeta Potential, and Surface Group Analysis of USPIO-101

**Figure 1:**
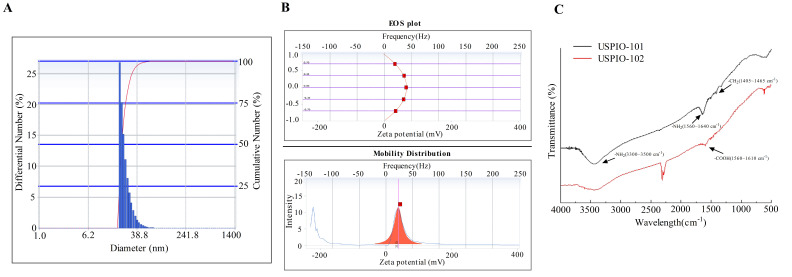
The dynamic light scattering (DLS), zeta potential analysis, and Fourier transform infrared spectroscopy (FTIR) spectra of ultrasmall magnetic iron oxide nanoparticles. (**A**) number distribution of USPIO-101, as measured by DLS. The concentration of USPIO-101 was 1 mg/mL. (**B**) Zeta potential analysis of USPIO-101. (**C**) Fourier transform infrared (FTIR) spectra are presented for USPIO-101 (black) and USPIO-102 (red), respectively. The -NH_2_ and -CH_2_ group signals are expressed in USPIO-101 at the wavelength of 3300~3500 cm^−1^, 1560~1640 cm^−1^, or 1405~1465 cm^−1^ (arrow). The -COOH group signal is expressed in USPIO-102 at the wavelength of 1550~1610 cm^−1^ (arrow).

Before the following experiments, the commercialized USPIO-101 measured its particle size, zeta potential, and surface group (Figure 1). The hydrodynamic diameter of USPIO-101 was 63.3 ± 2.3 nm; polydispersity index was 0.43 ± 0.58; zeta potential was 36.8 ± 0.6 mV (triple measurements, mean ± standard error, Figure S1). Next, we measured the surface group of USPIO-101 via FTIR. The spectra of FTIR represented that the –NH_2_ group expressed in the surface of USPIO-101 at the wavelength of 3300~3500 cm^−1^ or 1560~1640 cm^−1^. In addition, we also measured the positive control of the surface group –COOH at the wavelength of 1550~1610 cm^−1^ (Figure 1C). This result revealed that USPIO-101 majorly expressed –NH_2_ surface group.

### 3.2. USPIO-101 Alleviated the Allodynia Behavior via Intrathecal or Intraplantar Injection in Mice with Chronic Inflammatory Pain

**Figure 2:**
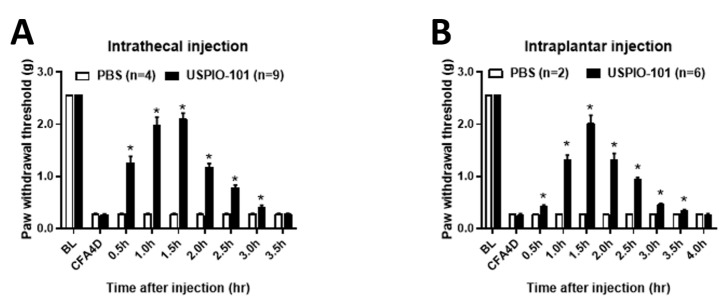
USPIO-101 attenuated chronic inflammatory pain via intrathecal or intraplantar injection. Mechanical pain sensitivity was measured using von Frey microfilaments. USPIO-101 attenuated the analgesia behavior in both (**A**) intrathecal and (**B**) intraplantar injection (10 mg/mL, 10 μL) after CFA paw injection for four days. The paw withdrawal thresholds were measured every 30 min until there was no difference between the three groups. Data were analyzed by two-way ANOVA and post-hoc with Tukey’s test. *: *p* < 0.05 vs. PBS sham group.

The analgesic effect of USPIO-101 was measured in mice with chronic inflammatory pain via different administration routes, intrathecal or intraplantar injection. After paw injection of CFA for four days, the mice showed decreased paw withdrawal thresholds. Both intrathecal (Figure 2A, PBS: 0.28 ± 0.02, USPIO-101: 2.1 ± 0.16 at 1.5 h, *p* < 0.05, two-way ANOVA and Bonferroni’s multiple comparisons) and intraplantar (Figure 2B, PBS: 0.29 ± 0.0, USPIO-101: 2.0 ± 0.15 at 1.5 h, *p* < 0.05, two-way ANOVA and Bonferroni’s multiple comparisons) injection of USPIO-101 (10 mg/mL, 10 μL) attenuated paw withdrawal thresholds.

### 3.3. USPIO-101 Decreased the Spinal Cord LTP at Spinal Cord Superficial Dorsal Horn Synapses in Mice with Chronic Inflammatory Pain and Naïve Mice

**Figure 3:**
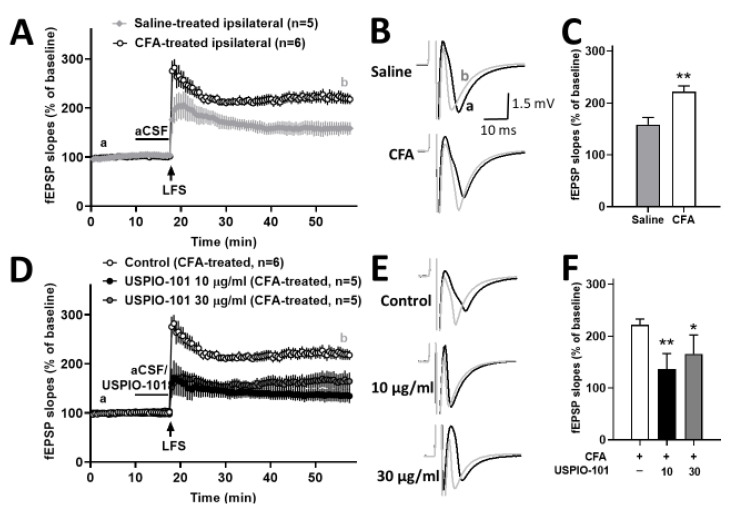
The effect of USPIO-101 on spinal cord LTP in CFA paw-injected mice. (**A**) Time courses of the slope of fEPSPs recorded before and after low-frequency stimulation (LFS, arrow) in spinal cord slices from CFA- or saline-treated mice. (**C**) The bar graph represents the magnitudes of potentiation, which averaged 20 fEPSPs recorded 30~40 min after LFS. **: *p* < 0.01 (unpaired *t*-tests) vs. CFA-treated ipsilateral group. (**D**) Time courses of the slope of fEPSPs recorded before and after LFS (arrow) in spinal cord slices from CFA-treated mice with USPIO-101 (10 or 30 µg/mL), respectively. USPIO-101 (10 or 30 µg/mL) applied for 7.5 min before LFS stimulation, respectively. (**F**) The bar graph represents the magnitudes of potentiation, which averaged 20 fEPSPs recorded 30~40 min after LFS at a concentration of 10 or 30 µg/mL. * or **: *p* < 0.05 or *p* < 0.01 vs. control group (one-way ANOVA). (**B**,**E**) Twenty recorded fEPSPs at time points a and b were averaged in each group. The slope of each fEPSP was expressed as % of the baseline fEPSP slope, which was the average of 20 fEPSPs at the beginning of 10 min recording. n indicates the number of slices recorded.

The potentiated spinal cord LTP at the spinal cord superficial dorsal horn synapses could stand for hyperalgesia [21]. The mice with chronic inflammatory pain showed the potentiated spinal cord LTP at the spinal cord superficial dorsal horn synapses was significantly higher than the control group (*p* < 0.05, *t*-test vs. saline-treated ipsilateral) (Figure 3A,C).

The concentration effect of USPIO-101 was tested on the LFS-evoked LTP at the superficial spinal dorsal horn in the spinal cord slices of mice with chronic inflammatory pain. USPIO-101 (10 or 30 µg/mL) was applied 7.5 min before LFS induction. The magnitude of LTP was significantly decreased in the treatments of 10 or 30 µg/mL (*p* < 0.01 or *p* < 0.05, one-way ANOVA vs. control), as shown in Figure 3D~3F. However, there was no concentration-dependent effect between 10 or 30 µg/mL (*p* > 0.05, *t*-test, 10 µg/mL vs. 30 µg/mL).

**Figure 4:**
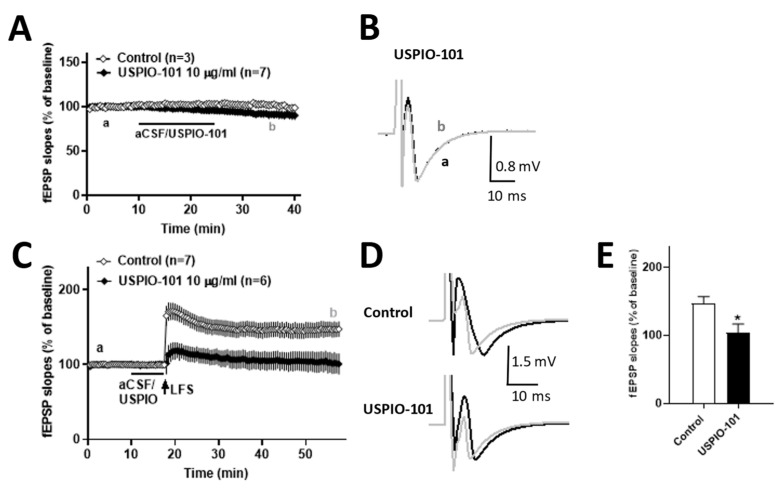
The effect of USPIO-101 on basal transmission (**A**) and spinal cord LTP (**B**,**C**) at the spinal cord superficial dorsal horn synapses, respectively. (**A**) After baseline recording for 10 min, USPIO-101 (10 µg/mL) was applied for another 15 min, then washed for 15 min. No difference was observed in the basal transmission after application of USPIO-101 to the spinal cord superficial dorsal horn synapses. (**C**) Time courses of the slope of fEPSPs recorded before and after LFS (arrow) in spinal cord slices. Drugs: USPIO-101 (10 µg/mL) was applied for 7.5 min before LFS stimulation. (**E**) The bar graph represents the magnitudes of potentiation, which averaged 20 fEPSPs recorded 30~40 min after LFS. *: *p* < 0.05 vs. control group (one-way ANOVA). (**B**,**D**) The traces shown in the graph are averaged 20 recordings of fEPSPs at times a and b in each group. The expression and analysis of the baseline fEPSP slope are the same as Figure 2. n indicates the number of slices recorded.

In the other part, we also tested the analgesic effect of USPIO-101 in naïve mice with the basal transmission or LFS-evoked LTP at the superficial spinal dorsal horn slices. No difference was observed in the basal transmission of naïve mice spinal cord slices when 10 µg/mL USPIO-101 was applied for 15 min (Figure 4A,B). However, USPIO-101 significantly inhibited the LTP in naïve mice spinal cord slices (Figure 4C,E). For USPIO-101 (10 µg/mL) applied 7.5 min before LFS induction, the magnitude of LTP significantly decreased in the treatment of USPIO-101 when compared with the control group (*p* < 0.05, Figure 4E).

These results revealed that USPIO-101 had an analgesic effect on inflammatory pain in the spinal cord through electrophysiological evidence.

### 3.4. Effects of USPIO-101 on Intracellular ROS Levels

**Figure 5:**
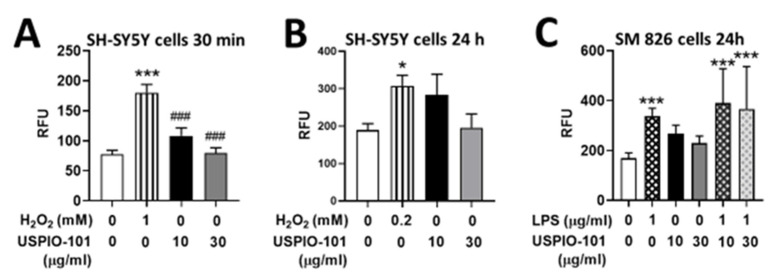
Intracellular reactive oxygen species level after treatment with USPIO-101 in SH-SY5Y or SM826 cells. (**A**,**B**) Hydrogen peroxide (H_2_O_2_) as positive control and USPIO-101 (10 or 30 μg/mL) were used in SH-SY5Y cells for 30 min (**A**, H_2_O_2_ 1 mM) and 24 h (**B**, H_2_O_2_ 0.2 mM), respectively. (**C**) Lipopolysaccharide (LPS) 1 μg/mL as positive control and USPIO-101 (10 or 30 μg/mL) were used in SM826 cells for 24 h, respectively. RFU means the relative fluorescence unit in 3 × 10^4^ cells/well in 96-well plates. Data shown are the mean ± SEM of three independent experiments performed in triplicate. * or ***: *p* < 0.05 or *p* < 0.001 vs. control group. ###: *p* < 0.001 vs. the H_2_O_2_ group (one-way ANOVA).

The iron oxide nanoparticle penetrates the cell and produces ROS [22], and the elevation of ROS induces neurotoxicity [23]. The ROS production ability of USPIO-101 was measured in neuron-like or microglial cells (SH-SY5Y or SM826 cells). Hydrogen peroxide (H_2_O_2_) was used as a positive control in SH-SY5Y cells for short (1 mM, 30 min) or long (0.2 mM, 24 h) stimulation, and compared to two USPIO-101 concentrations (10 or 30 μg/mL) as shown in Figure 5A,B. The intracellular ROS levels were only significantly increased in the group of H_2_O_2_ (*p* < 0.05, one-way ANOVA, vs. control, Figure 5A,B). In SM826 cells, USPIO-101 (10 or 30 μg/mL) did not induce significant elevation of ROS when compared with the control (*p* < 0.05, one-way ANOVA, vs. control, Figure 5C). The ROS level of co-treatment of LPS and USPIO-101 was close to the group of LPS alone (positive control, *p* > 0.05, one-way ANOVA, vs. LPS, Figure 5C) and significantly higher than the control (*p* < 0.05, one-way ANOVA, vs. control, Figure 5C).

These results suggested that USPIO-101 did not elicit ROS toxicity in neuron-like or microglial cells.

### 3.5. Effects of USPIO-101 on mRNA Expression with LPS Stimulation

**Figure 6:**
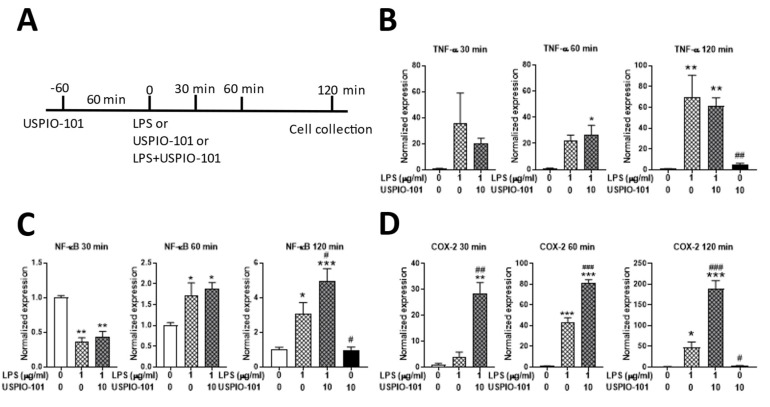
USPIO-101 potentiates the LPS-induced NF-kB and COX-2 mRNA expression in SM826 microglial cells. (**A**) The time scale for treating USPIO-101 and LPS stimulation. (**B**–**D**) The levels of mRNAs encoding the pro-inflammatory cytokines TNF-α (**B**), NF-κB (**C**), and COX-2 (**D**) were analyzed by qRT-PCR and normalized by the expression of GAPDH (glyceraldehyde-3-phosphate dehydrogenase) at three time points after LPS stimulation: 30, 60, or 120 min. Data are presented as mean ± SEM (*n* = 4). * or ** or ***: *p* < 0.05, or *p* < 0.01, or *p* < 0.001 vs. control; # or ## or ###: *p* < 0.05, or *p* < 0.01, or *p* < 0.001 vs. LPS.

To examine the effect of USPIO-101 on the pro-inflammatory cytokines’ mRNA expression with LPS stimulation, the RT-qPCR was used for measuring mRNA extracted from microglia cells. USPIO-101 (10 μg/mL) was pretreated for 1 h before LPS (1 μg/mL) stimulation, and the cells were collected after LPS stimulation for 30, 60, or 120 min. LPS treatment for 120 min significantly upregulated the levels of transcripts encoding the pro-inflammatory cytokines TNF-α, NF-κB, and COX-2 (*p* < 0.05 vs. control, one-way ANOVA, Figure 6B–D). USPIO-101 significantly enhanced the expression of NF-κB and COX-2 after LPS stimulation for 120 min (*p* < 0.05 vs. LPS, one-way ANOVA, Figure 6C,D). USPIO-101 significantly enhanced the mRNA expression of COX-2 in the group of LPS + USPIO-101 after LPS 30-, 60-, or 120-min treatment (*p* < 0.05 vs. LPS, one-way ANOVA, Figure 6D). USPIO-101 treatment alone, for 3 h, did not affect the mRNA expression of these pro-inflammatory cytokines (Figure 6). These results suggest that USPIO-101 enhances the mRNA expression of pro-inflammatory cytokines, especially COX-2, when co-treated with LPS stimulation in the microglia cells.

### 3.6. USPIO-101 Impaired the Hippocampal LTP at the Schaffer Collateral-CA1 Synapses

The effect of USPIO-101 on the hippocampal LTP is still unknown. Here, we investigated whether the hippocampal LTP at the Schaffer collateral-CA1 synapses was affected by USPIO-101 in naïve mice.

**Figure 7:**
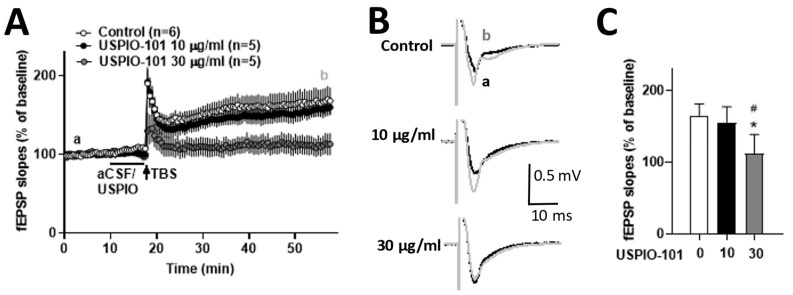
The effect of USPIO-101 on hippocampal LTP. (**A**) Time courses of the slope of fEPSPs recorded before and after theta-burst stimulation (TBS, arrow) in hippocampal slices. Drugs: USPIO-101 (10 or 30 µg/mL) was applied for 7.5 min before TBS stimulation. (**B**) The traces shown in the graph are the average of 20 recorded fEPSPs at times a and b in each group. (**C**) The bar graph represents the magnitudes of potentiation, with the average of 20 fEPSPs recorded 30~40 min after TBS at a concentration of 10 or 30 µg/mL. *: *p* < 0.05 vs. control group. #: *p* < 0.05 vs. USPIO-101 group (one-way ANOVA). n indicates the number of slices recorded. The expression and analysis of the baseline fEPSP slope are the same as Figure 3.

The concentration effect of USPIO-101 was elucidated in the hippocampal slice, and USPIO-101 (10 or 30 µg/mL) was applied 7.5 min before TBS induction. The magnitude of LTP was not affected at 10 µg/mL, but significantly decreased in the treatment of 30 µg/mL (*p* < 0.01, one-way ANOVA vs. control, Figure 7). These results revealed that USPIO-101 could impair hippocampal LTP at a higher concentration, and suggested the neurotoxicity possibility of USPIO-101.

## 4. Discussions

Our results showed the dual effect of USPIO-101: one effect was the alleviation of inflammatory pain at the spinal cord; the other was the risk of neurotoxicity.

### 4.1. The Analgesic Effect of USPIO-101

Our results showed USPIO-101 (10 mg/mL, 10 µL) increased the mechanical paw withdrawal thresholds through intrathecal or intraplantar injection. Comparing intrathecal to intraplantar injection, intrathecal injection had the higher paw withdrawal thresholds at time 0.5 h, which suggested intrathecal injection was more potent than intraplantar injection at the onset time.

The in vitro electrophysiological study showed that USPIO-101 (10 or 30 µg/mL) partially decreased the potentiated spinal LTP at the spinal superficial dorsal horn synapses in mice with chronic inflammatory pain. The USPIO-101-treated spinal LTP level in CFA-treated mice was almost back to the level of saline-treated spinal LTP (Figure 3D). However, the LFS-induced potentiated spinal LTP in naïve mice could be entirely abolished by USPIO-101 (10 µg/mL) (Figure 4C). In the disease (inflammatory pain) model, the potentiated spinal LTP was more complicated than the naïve state. Even at higher concentrations, the potentiated spinal LTP would be no further decreased by USPIO-101 (30 µg/mL). The reasons why USPIO-101 only partially reduced the LFS-induced LTP in mice with chronic inflammatory pain are still unknown. However, this maintained spinal LTP in CFA-treated mice was probably why USPIO-101 only attenuated the inflammatory pain for a short duration (less than 3.5 h, Figure 2) in the behavioral tests.

The activity-dependent effect was another character of USPIO-101 revealed from our data. USPIO-101 did not affect the basal transmission but inhibited the LFS-evoked spinal LTP (Figure 4). The LFS-evoked LTP was associated with the injury or inflammatory situation at the spinal cord superficial dorsal horn synapses [24]. These results demonstrate the analgesic effect of USPIO-101 on inflammatory pain, which has been reported in our previous study [5,25] and our present study (Figure 2).

### 4.2. Cell Toxicity of USPIO-101

The iron oxide nanoparticles were less toxic than other ion nanoparticles, especially USPIO [26]. However, more evidence revealed that USPIO could induce cytotoxicity due to the size, shape, surface charge, or coating of the nanoparticles [27]. The physicochemical character of USPIO-101 showed that USPIO-101 probably had some aggregation with the self or other ions, but kept normal stability. Meanwhile, although USPIO-101 has proven analgesia ability, the cytotoxicity of USPIO-101 is less known.

Our data showed that USPIO-101 (10 or 30 µg/mL) did not induce the significant elevation of ROS in SH-SY5Y or SM826 cells for 30 min or 24 h, compared with the positive control (Figure 5). However, after the treatment of USPIO-101 for 24 h, the production of ROS was still high at the concentration of 10 µg/mL in a trend (SH-SY5Y or SM 826 cell: *p* = 0.07 or *p* = 0.014, control vs. USPIO-101 10 µg/mL 24 h, unpaired *t*-test). This suggested that USPIO-101 could induce ROS production with a chronic but not acute effect in neuron-like or microglial cells.

Our mRNA data showed that COX-2 was significantly up-regulated after LFS stimulation for 30~120 min in microglial cells (Figure 6D). COX-2 was an enzyme involved in synthesizing prostaglandins (PGs), and the induction of COX-2 enhanced nociception via increasing PG release [28]. This was controversial to our analgesic results. However, the spinal cord and brain microglia had a different response to inflammatory stimulation for unknown reasons [29]. The SM826 cell was derived from the brains of mice [30], which probably could not represent the actual situation in the spinal cord. Our data suggested that USPIO-101 could likely induce the elevation of COX-2 in brain microglial cells, but not the spinal cord.

The cytotoxicity of USPIO has been reported [9,10,11,12], but less is known about COX-2. Only one study has revealed that COX-2 is elevated after SPIO treatment in the liver [11]. We still do not know why USPIO-101 induces COX-2 so quickly during LPS stimulation in the microglial cells, because phagocytic cells, such as microglia cells, are not as sensitive to positive surface charge nanoparticles as they are to negative surface charges [31].

### 4.3. Hippocampal LTP Was Impaired by USPIO-101 at a Higher Concentration

We measured the effect of USPIO-101 on the hippocampal LTP to predict if USPIO-101 has neurotoxicity in the hippocampus. Our data demonstrated USPIO-101 could impair hippocampal LTP at a higher concentration (Figure 7). Another study revealed that USPIO could induce neurotoxicity in the hippocampus via in vivo study. A direct single injection of USPIO (1 µg/µL, size: 30 nm) into the mouse hippocampus for 7 or 14 days could impair the animals’ spatial memory in the Morris water maze test [32]. Other controversial data showed no toxicity response to intranasally instilled Fe_3_O_4_ (1 mg/mL, size: 30 nm) nanoparticles in the brain [33]. One possible explanation of neurotoxicity in the hippocampus was that hippocampal neurons were more sensitive to SH-SY5Y cells when applied with exogenous iron, showing higher cell death [34].

Our data showed USPIO-101 could impair hippocampal LTP, which suggests that USPIO-101 probably has toxicity in hippocampal neurons or can antagonize some ionic receptors which are essential for early LTP induction, such as the N-methyl-D-aspartate (NMDA) receptor [35], the α-amino-3-hydroxy-5-methyl-4-isoxazolepropionic acid (AMPA) receptor [36], or other targets. More evidence is needed to elucidate this.

### 4.4. The Effect of Surface Group

Our used TANBead^®^ USPIO-101 was a conventional product designed to conjugate with target-specific molecules through the amide-bond formation with carbodiimide-activated carboxylic acid groups. We investigated another carboxyl group product from the same company, TANBead^®^ USPIO-102, on the analgesic effect of hippocampal neurotoxicity. However, we observed that USPIO-102 had a less analgesic response and no neurotoxicity in the hippocampal slice, compared with USPIO-101 (Figures S2 and S3). The properties of USPIO-101 and USPIO-102 are almost the same, including size (6~10 nm), solvent (water), and stock concentration (10 mg/mL). The only different parts are the surface, amine group (USPIO-101), or carboxyl group (USPIO-102), as shown in Figure 1C. The surface group for Fe_3_O_4_ nanoparticles is critical, because naked Fe_3_O_4_ has a high surface energy, leading to aggregation and oxidation [37]. In addition, both amine and carboxyl groups are hydrophilic groups, which strongly attract water solubility, good biological compatibility, and biodegradability [38]. However, the physiological function of the different surfaces is still unclear.

## 5. Conclusions

Our results revealed the dual effect of USPIO-101: it could relieve inflammatory pain at the spinal cord, but also induce neurotoxicity in the central brain. These localized administration routes (e.g., intrathecal or intraplantar administration) of USPIO-101 did not elicit a toxicity response during our experiments; however, if the USPIO-101 leaks to the brain, it would probably impair hippocampal LTP at higher concentrations.

## Data Availability

Not applicable.

## Supplementary Materials

The following supporting information can be downloaded at: https://www.mdpi.com/article/10.3390/pharmaceutics14020366/s1, Figure S1: the dynamic light scattering (DLS) and zeta potential analysis of USPIO-101. Figure S2: USPIO-101 and USPIO-102 attenuate chronic inflammatory pain via intrathecal or intraplantar injection. Figure S3: The effect of USPIO-101 or USPIO-102 on hippocampal LTP, respectively.
