# Supplementary material for: The Surface Amine Group of Ultrasmall Magnetic Iron Oxide Nanoparticles Produce Analgesia in the Spinal Cord and Decrease Long-Term Potentiation
Source: Pharmaceutics. 2022 Feb 6;14(2):366. doi: 10.3390/pharmaceutics14020366 (PMC8879719; doi:10.3390/pharmaceutics14020366)
Supplement: Supplementary file 1 [file pharmaceutics-14-00366-s001.zip › pharmaceutics-1533233-Supplementary.pdf]

pharmaceutics

MDPI

Supplementary Materials: The Surface Amine Group of Ultrasmall Magnetic Iron Oxide Nanoparticles Produce Analgesia in the Spinal Cord and Decrease Long-Term Potentiation

Guan-Ling Lu, Ya-Chi Lin, Ping-Ching Wu and Yen-Chin Liu

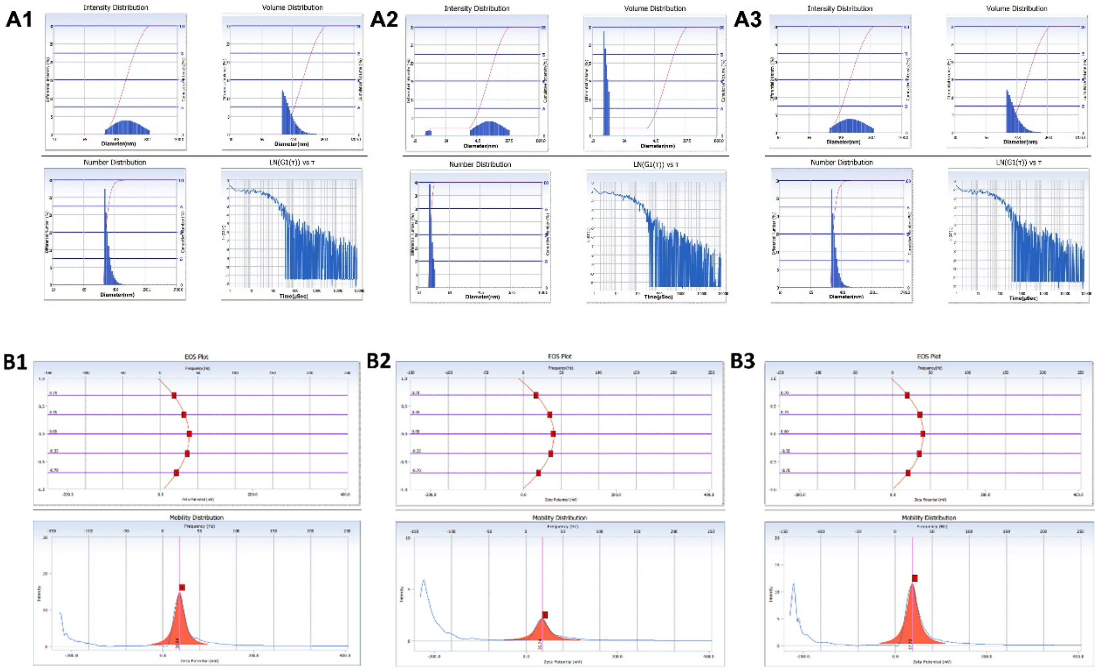

Figure S1. the dynamic light scattering (DLS) and zeta potential analysis of USPIO-101. (A) Intensity, volume, and number distribution and autocorrelation function of DLS. The concentration of USPIO-101 was 1 mg/ml. **(B)** zeta potential analysis of USPIO-101. The electrosmosis (EOS) plot and the zeta potential distribution graph for a flat solid surface were shown. A1~A3 and B1~B3 represented triple measurements.

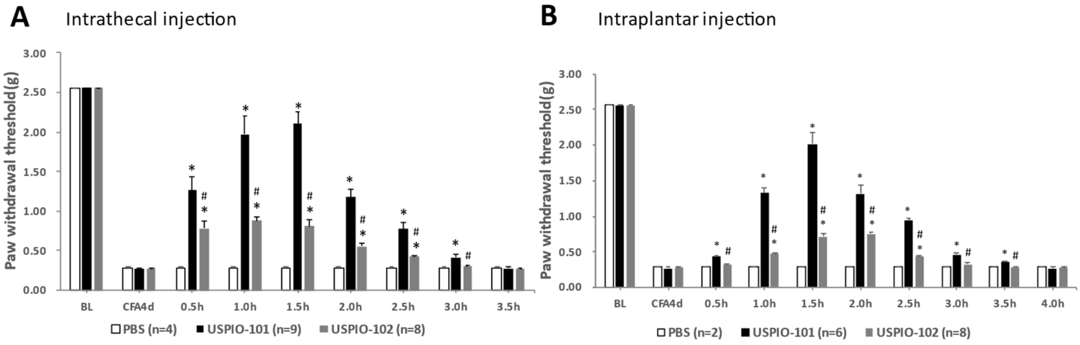

Figure S2. USPIO-101 and USPIO-102 attenuate chronic inflammatory pain via intrathecal or intraplantar injection. Mechanical pain sensitivity was measured using von Frey microfilaments. USPIO-101 and USPIO-102 attenuate the analgesia behavior in both (A) intrathecal and (B) intraplantar injection (10 mg/ml, 10 μl) after CFA paw injection for 4 days. The paw withdrawal thresholds were measured every 30 min until there was no difference between the three groups. Data

were analyzed by two-way ANOVA and post-hoc with Tukey test. *: p<0.05 vs. PBS sham group; #: p<0.05 vs. USPIO-101 group.

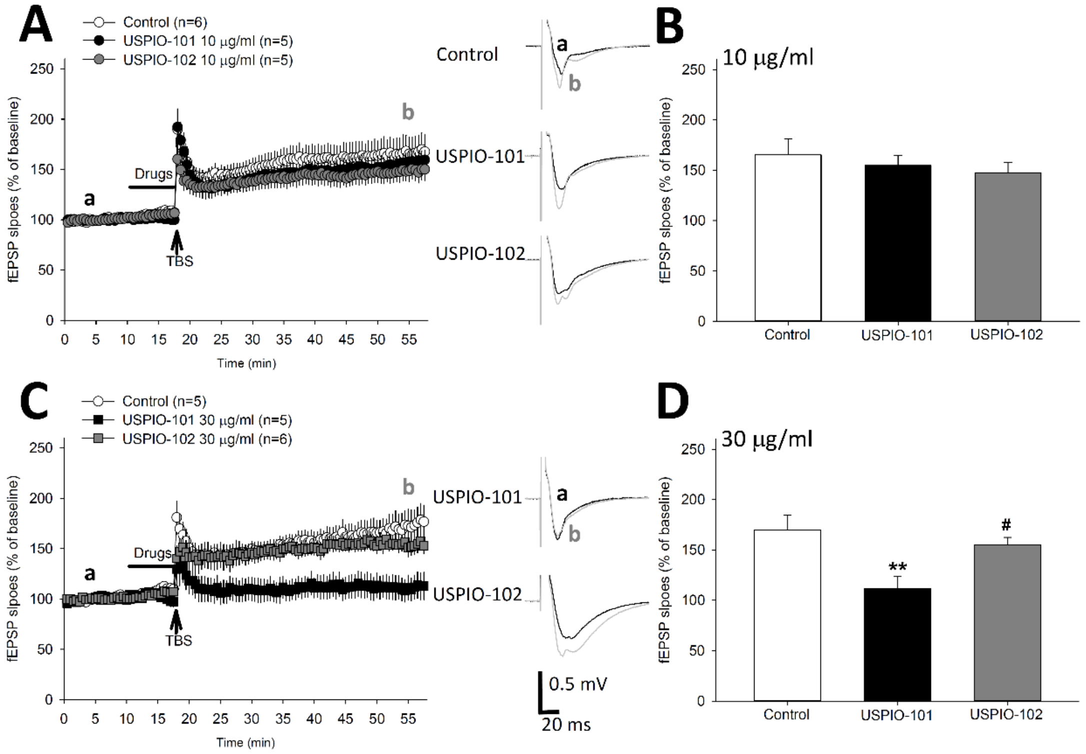

Figure S3. The effect of USPIO-101 or USPIO-102 on hippocampal LTP, respectively. (A and C) Time courses of the slope of fEPSPs recorded before and after theta-burst stimulation (TBS, arrow) in hippocampal slices. Drugs, USPIO-101 or USPIO-102 (10 or 30 μg/ml) was applied for 7.5 min before TBS stimulation, respectively. **(B and D)** The bar graph represented the magnitudes of potentiation, which averaged 20 fEPSPs recorded 30~40 min after TBS at a concentration of 10 μg/ml (B) or 30 μg/ml (D). The bar graph represented the magnitudes of potentiation, which averaged 20 fEPSPs recorded 30~40 min after TBS. The slope of each fEPSP was expressed as % of the baseline fEPSP slope, which was the average of 20 fEPSPs at the beginning of 10 min recording. Twenty recorded fEPSPs at time points a and b were averaged in each group, as shown within the graph. One-way ANOVA was used for statistical analysis. **: p<0.01 vs. control group. #: p<0.05 vs. USPIO-101 group. n indicates the number of slices recorded.
